# The importance of good history taking: a case report

**DOI:** 10.1186/s13256-015-0559-y

**Published:** 2015-04-30

**Authors:** Durga Ghosh, Premalatha Karunaratne

**Affiliations:** 1 Acute Medicine Registrar, Dumfries and Galloway Royal Infirmary, Bankend Road, Dumfries, DG1 4AP UK; 2 Vale of Leven District General Hospital, Main Street, Alexandria, G83 0UA UK

**Keywords:** Older adult, Comprehensive geriatric assessment, Neurological examination, Stroke, Vasculitic neuropathy

## Abstract

**Introduction:**

Early comprehensive geriatric assessment (CGA) with good history-taking is essential in assessing the older adult.

**Case presentation:**

Our patient, a 75-year-old Caucasian woman, was originally admitted to hospital for investigation of iron deficiency anemia. During admission, she developed pneumonia and new intermittent atrial fibrillation in association with a right-sided weakness, which was felt to be new at the time.

Following this episode, she was treated for a further chest infection and, despite clinical improvement, her inflammatory markers failed to settle satisfactorily.

She was transferred to her local hospital for a period of rehabilitation where further neurological findings made the diagnosis of solely stroke questionable; these findings prompted further history-taking, investigations and input from other disciplines, thereby helping to arrive at a working diagnosis of vasculitic neuropathy.

**Conclusions:**

The case aims to highlight the importance of taking a good history and performing an early comprehensive assessment in the older adult.

## Introduction

Good history-taking, an essential part of a comprehensive assessment in an older adult [1], helped reveal an underlying debilitating neuropathy.

## Case presentation

Our patient, a 75-year-old Caucasian woman, was admitted to hospital for investigation of iron deficiency anemia in June 2013. Her hemoglobin (Hb), hematocrit and mean cell volume (MCV) levels preadmission were 10.1g/dL, 0.33 and 77 fL, respectively.

There was little known about her past medical history aside from type 2 diabetes mellitus (T2DM) requiring insulin, hypertension and chronic obstructive pulmonary disease (COPD). Investigation for iron deficiency anemia confirmed extensive diverticular disease with normal upper gastrointestinal (GI) endoscopy and duodenal biopsy.

During the same admission, she developed a hospital-acquired pneumonia and new intermittent atrial fibrillation.

Coinciding with this period, she developed a new dysphasia and what was perceived to be a ‘new’ right-sided weakness. A computed tomography (CT) brain scan showed no acute change and she was treated as a patient with ischemic stroke, given the clinical findings.

She was treated for a further pneumonia in hospital and also underwent investigations such as a CT pulmonary angiography (CTPA) scan, which ruled out pulmonary embolism but confirmed partial left lung collapse; subsequent bronchoscopy was negative for malignancy. Her inflammatory markers remained elevated with her C-reactive protein (CRP) level approximately 140 and white cell count (WCC) 14 but she remained mentally alert and made clinical improvement. Her repeat chest X-rays were also unchanged. Given the clinical improvement, she was deemed suitable for transfer for stroke rehabilitation to her local hospital in August 2013. Her medications on transfer were: Novomix 30 twice daily (later stopped due to low blood sugar levels); clopidogrel 75 mg; quinine sulphate 200 mg; ranitidine 150 mg twice daily; folic acid 5mg; and digoxin 125mcg.

In the Rehabilitation and Assessment Directorate (RAD), the assumption was that our patient had suffered a stroke causing a right-sided weakness, as per the handover pre-transfer, however, further neurological features were detected on the post-take ward round as listed below: right lower motor neurone seventh nerve weakness; ptosis right greater than left; bilateral wrist drop; bilateral foot drop; generalized reduced tone and reduced power in all four limbs: right arm 3 out of 5, right leg 0 out of 5, left arm 3 to 4 out of 5, and left leg 2 out of 5. No comment was documented regarding sensation.

The chronicity of the neurological features was uncertain at this point as they did not appear to have been previously documented and the immediate reaction was to exclude an acute neurological process. Fortunately, her daughter was present during the ward round that day and a collateral history revealed our patient had ‘possibly’ been like this for 18 months or more. This led to a degree of reassurance with regard to the fact that the neurological findings were unlikely to be acute.

Further discussion with our patient’s son confirmed that her mobility had gradually deteriorated over a two-year period; from his perception, the only novel finding was alteration of our patient’s speech at the time of a presumed ischemic stroke.

Given the account from our patient’s son and daughter, her general practitioner (GP) was also contacted and it was reported that our patient had been referred to various disciplines for poor mobility but had failed to attend her appointments; she had been diagnosed with a Bell’s palsy and third nerve palsy in 2011, which had been attributed to her diabetes. Interestingly, a previous trial of steroids from her GP for presumed arthropathy had resulted in clinical improvement.

Our patient underwent several investigations, included below, as part of the investigative and diagnostic process. In addition, for the complete history, her blood pressure was 104/53 mmHg and heart rate was 57 beats/minute with no documented murmurs.

### Imaging

A magnetic resonance imaging (MRI) brain scan showed atrophy with small vessel disease, high signal at the left corona radiata and adjacent left occipital horn. An MRI cervical spine scan revealed no gross abnormality and a CT scan of her abdomen and pelvis showed extensive diverticular disease only.

### Blood investigations

Blood tests showed her Hb level was 85g/dL; WCC was 11.1; platelets were 512, MCV was 89 and her hematinics were normal. Urea and electrolytes test (U&E), liver function test (LFT), and calcium test results were normal. Her albumin level was l0, CRP level was 120 and her baseline HBA1c level was 57mmol/L (normal range 20 to 42mmol/L). Her cholesterol level was unavailable and her blood and urine cultures were negative.

#### Vasculitic screen

Her antinuclear antibody test (ANA) results were 1/40 and showed a speckled pattern. She had low C3 0.71g/L (0.88 to 1.82g/L) and low C4 0.08g/L (0.16 to 0.45g/L). Her rheumatoid factor was 623, antineutrophil cytoplasmic antibody (cANCA) results were strongly positive and myeloperoxidase**/**proteinase 3 (MPO/PR3) negative. Her immunoglobulin A (IgA) level was 6.5, erythrocyte sedimentation rate (ESR) 115, and cryoglobulins were negative. Her carcinoembryonic antigen (CEA) and serum angiotensin-converting enzyme (ACE) levels were normal, and CA 125 test result was 70 (0 to 35). Serum electrophoresis showed no paraproteins and her neuroimmunological blood test results were negative.

#### Invasive investigations

Her lumbar puncture test result was negative.

#### Neurology unit investigations

**Electromyography** (EMG) showed severe axonal sensory motor neuropathy.

Multi-specialist input from several disciplines including rheumatology and neurology was also requested.

The neurologist documented the following clinical findings: right ptosis; right facial weakness; generalized weakness right greater than left; profound distal greater than proximal weakness and wasting left greater than right; upper limb greater than lower limb; sensation distally decreased in lower limbs; and areflexia.

The general consensus was that our patient was probably manifesting a peripheral neuropathy secondary to a vasculitis (the type of which was difficult to classify); the neuropathy had been possibly exacerbated by a recent stroke; the stroke may have been part of the vasculitic process itself or could have been related to atrial fibrillation.

It was felt that a nerve biopsy would have little else to contribute to the diagnosis and simultaneously might induce patient distress and was therefore avoided.

Given her history of T2DM, our patient was cautiously commenced on a trial of prednisolone 40mg with azathioprine on 13 September 2013; additional oral antidiabetic therapy on advice of the diabetic team was commenced and gradual step-down of steroid therapy was planned.

Within days of steroid initiation, our patient’s inflammatory markers improved. Her CRP level fell to 36 and her WCC to single figures.

Her right wrist drop showed slight improvement from initial investigations to the point of discharge. There was, however, little neurological and functional improvement otherwise.

Our patient remained bedbound and her hospital stay was complicated by a pressure ulcer, which had completely healed prior to discharge to a care home six months later. On discharge her blood test results revealed Hb of 94, WCC of 11.5, her platelets were 380 and MCV 90.8, her CRP level was 20 and albumin level was 28. She remained clinically stable and her treatment goal was to help prevent any further neurological deterioration.

## Discussion

Our patient had been admitted for investigation of iron deficiency anemia and suffered recurrent illness during admission precipitating a prolonged hospital admission and eventual transfer to her local hospital for stroke rehabilitation.

Looking back at the case, our patient did have a stroke as was confirmed on MRI; however, the fact that she had bilateral and long-standing neurological signs evaded detection for a considerable period of time.

It is only after a thorough history-taking, examination and comprehensive geriatric assessment post transfer to the rehabilitation unit that her illness was diagnosed. Though there may not have been a great change to her overall quality of life, an underlying debilitating diagnosis was established with a treatment goal attempting to prevent further neurological deterioration.

It can be argued that had a comprehensive geriatric assessment taken place earlier, keeping in mind that she had displayed neurological symptoms some 18 months previously, would earlier initiation of treatment been more effective in improving her quality of life? Should the fact that she had failed to attend appointments prompted further thinking as to the underlying factor causing nonattendance at clinic appointments, keeping in mind that there was a history of neurological signs?

## Conclusions

The case aims to highlight the importance of taking a good history and performing a comprehensive assessment, especially in the older adult [1].

## Consent

Written informed consent was obtained from the patient’s daughter for publication of clinical details and this case report. The patient verbally consented to publication of the case report but was unable to sign the document due to her wrist drop. A copy of the written consent is available for review by the Editor-in-Chief of this journal.

## Abbreviations

ACE: angiotensin-converting enzyme
ANA: antinuclear antibody
ANCA: antineutrophil cytoplasmic antibody
CEA: carcinoembryonic antigen
COPD: chronic obstructive airways disease
CRP: C- reactive protein
CT: computed tomography
CTPA: CT pulmonary angiogram
EMG: electromyography
ESR: erythrocyte sedimentation rate
GI: gastrointestinal
GP: general practitioner
Hb: hemoglobin
IgA: immunoglobulin A
LFT: liver function test
MCV: mean cell volume
MPO/PR3: myeloperoxidase/proteinase 3
MRI: magnetic resonance imaging
RAD: Rehabilitation and Assessment Directorate
T2DM: type 2 diabetes mellitus
U&E: urea and electrolytes test
WCC: white cell count
